# PP73 Evaluating Alignment Between FDA Accelerated Approvals And EMA, MHRA, And NICE Recommendations

**DOI:** 10.1017/S0266462325102912

**Published:** 2025-12-29

**Authors:** Mouna Dardouri, Michael J. DiStefano

## Abstract

**Introduction:**

Accelerated approval facilitates timely access to innovative drugs in the USA, but confirmatory trials are required for traditional approval. This study evaluated alignment between United States Food and Drug Administration (FDA), European Medicines Agency (EMA), Medicines & Healthcare Products Regulatory Agency (MHRA), and National Institute for Health and Care Excellence (NICE) recommendations regarding drugs converted by the FDA from accelerated to traditional approval. This research addresses a gap in the literature by examining drugs across all therapeutic categories converted to traditional approval after 2020.

**Methods:**

This retrospective cohort study analyzed drug-indication pairs granted FDA accelerated approval and converted to traditional approval between January 2020 and October 2024. Data from EMA, MHRA, and NICE documents were reviewed to identify decisions regarding market authorization (EMA and MHRA) and coverage recommendation type (NICE). Drug-indication pairs were categorized by FDA therapeutic classification, including infectious diseases (vaccines and non-vaccines), cancer, hematologic, neurological, and other disorders.

**Results:**

From January 2020 to October 2024, 115 drug-indication pairs received FDA accelerated approval. Eight of these were later withdrawn and 78 remain with ongoing confirmatory trials, while 29 successfully verified clinical benefit and were converted to traditional approval. Among these, 25 received EMA market authorization and 21 received MHRA market access after departure from the European Union (EU), with 14 requiring additional monitoring. By October 2024, 14 pairs had not been reviewed by NICE. Eight drug-indication pairs received a “recommended” status, two were “optimized” for smaller populations, and two were recommended for the Cancer Drugs Fund. One appraisal was terminated, and two were not recommended due to insufficient clinical benefits or cost effectiveness.

**Conclusions:**

This study showed that FDA decisions to convert drugs from accelerated to traditional approval do not always align with EMA, MHRA, and NICE recommendations, highlighting potential global differences in regulatory and reimbursement criteria. Next steps will focus on analyzing NICE appraisal rationales, clinical evidence, and cost-effectiveness considerations to better understand these discrepancies, particularly in the post-EU departure period.

